# In Vitro Activity of *Foeniculum vulgare* and Its Main Essential Oil Component Trans-Anethole on *Trichomonas vaginalis*

**Published:** 2019

**Authors:** Faezeh KARAMI, Dara DASTAN, Mohammad FALLAH, Mohammad MATINI

**Affiliations:** 1. Department of Medical Parasitology and Mycology, School of Medicine, Hamadan University of Medical Sciences, Hamadan, Iran; 2. Medicinal Plants and Natural Products Research Center, Hamadan University of Medical Sciences, Hamadan, Iran; 3. Department of Pharmacognosy and Pharmaceutical Biotechnology, School of Pharmacy, Hamadan University of Medical Sciences, Hamadan, Iran

**Keywords:** Anethole, Essential oil, Extract, *Foeniculum vulgare*, *Trichomonas vaginalis*

## Abstract

**Background:**

Trichomoniasis is one of the most common nonviral sexually transmitted infections worldwide which drug-resistant cases of the infection are rising. The aim of the study was to assessment the in vitro activity of *Foeniculum vulgare* and its main essential oil component on *Trichomonas vaginalis*. Also phytochemical investigation of *F. vulgare* essential oil was performed.

**Methods:**

Five *T. vaginalis* isolates subjected to susceptibility testing against essential oil and extracts of *F. vulgare* and anethole using microtiter plate method. The minimum lethal concentration (MLC) of the natural products was assessed in comparison with metronidazole. Gas chromatography-mass spectrometry and gas chromatography-flame ionization detector was applied for chemical investigation of the essential oil.

**Results:**

After 48 hours incubation, the most potent antitrichomonal agents were the methanolic and hexanic extract with MLC of 360 μg/ml and followed by the essential oil and anethole (1600 μg/ml). The isolates were sensitive to metronidazole with a mean MLC of 13.7 μg/ml. E-Anethole (88.41 %) was the major constituent of *F. vulgare* essential oil.

**Conclusion:**

The results suggested in vitro antiprotozoal properties of *F. vulgare* and anethole against *T. vaginalis*. Therefore further studies are needed to evaluate their in vivo effects and toxicity.

## Introduction

Human trichomoniasis, caused by the protozoan parasite *Trichomonas vaginalis*, is the most prevalent nonviral sexually transmitted disease (STD) worldwide. The global prevalence of the infection is estimated 276.4 million cases annually that is more than other STDs such as gonorrhea, syphilis, and chlamydia infection. Adverse pregnancy outcomes, infertility, cervical neoplasia and increased risk of HIV transmission are known complications of the infection (1–3).

Since 1961, metronidazole, a 5-nitroimidazole derivative, has been used for treatment of trichomoniasis. Treatment failure with metronidazole was observed very soon in 1692 (4, 5). Resistant *T. vaginalis* isolates have been estimated up to 2 to 5% of clinical isolates in the United States. Therefore, increased refractory trichomoniasis is concerning because of few available alternative drugs (6, 7). Therefore, the evidence suggest the need for further research on management of trichomoniasis.

Most chemotherapy drugs have a natural source; hence, medicinal plant and their bioactive phytochemicals are of particular interest for pharmaceutical research. *Foeniculum vulgare* (Apiaceae), known as fennel, is indigenous to the Mediterranean area and has widely naturalized in many parts of the world. This medicinal and aromatic plant with the common Persian name of Razianeh, in addition to culinary uses, has been traditionally applied for the treatment of gastrointestinal and respiratory disorder. A number of bioactive compounds such as trans-anethole, fenchone, estragole, sesquiterpenoids, coumarins and polyphenolics have been identified in fennel (8, 9). Several studies have documented significant antimicrobial effects of *F. vulgare* and anethole against some fungal and bacterial species (10–14).

Given the antimicrobial activity of fennel, this research was conducted to evaluate the in vitro antiprotozoal activity of *F. vulgare* and trans-anethole, a member of the phenylpropanoids class of chemical compounds, on *T. vaginalis* parasite.

## Materials and Methods

### Plant and Chemicals

The prepared seeds of fennel were initially identified by Medicinal Plants and Natural Products Research Center, Hamadan University of Medical Sciences, Hamadan, Iran. Trans-anethole (800429) was purchased from Merck Chemicals Co. (Darmstadt, Germany) and Metronidazole (M3761) and Dimethyl sulfoxide (D2650, BioReagent) were obtained from Sigma-Aldrich Chemical Co. (St. Louis,. MO, U.S.A.).

### Extracts and essential oil preparation

The seeds of fennel were powdered and subjected to extraction by maceration method (15). In brief, the powdered seeds (100g) was macerated in three solvent: *n*-hexane, methanol and distilled water (3×1L, rt for 72 h), respectively. The mixtures were filtered to separate solid materials and then evaporated by a rotary evaporator under vacuum at below 40 °C. The essential oil was obtained from the powdered seed (120g) by hydrodistillation method for three hours extraction time using a Clevenger-type apparatus (16). Afterwards, the oil was dehydrated by anhydrous sodium sulfate. Finally, the obtained extracts and oil were stored in dark airtight container at 4 °C until use.

### Isolates and solutions

Susceptibility testing was done by five clinical *T. vaginalis* isolates (Tv3, Tv15, Tv16, Tv17, Tv18) that were previously grown in TYI-S-33 medium (17). Distilled water and dimethyl sulfoxide (DMSO) were used to dissolve metronidazole and the natural products, respectively.

### MLC, GI% and susceptibility testing

The minimum lethal concentration (MLC) shows the lowest concentration of the agents that caused immobilization and death of *T. vaginalis* trophozoites (18). The growth inhibitory percentage (GI%) indicates sublethal concentration (sub-MLC) that causes relative growth inhibition of *T. vaginalis* trophozoites (19).

Aerobic microplate susceptibility testing was done according to the Centers for Disease Control and Prevention's method (18). The duplicate tests were performed against negative control (medium without any antitrichomonal agent) and DMSO control under sterile condition and repeated three independent times. Briefly, the agents were subjected to two-fold serial dilutions with Diamond’s medium. Then, 100 μl of the parasite culture (1×10 ^5^ cells/ml) was added to the wells and incubated at 35.5 °C for 24 and 48 hours. Then, the microplates were inspected by inverted microscope and the lowest concentration of the agents in microplate well in which no motile trichomonads were detected, considered as MLC (17). To determine the GI% value, the trichomonads cells were counted using the haemocytometer (Neubauer cell-counter chamber) and GI% was obtained according to the equation: $\text{GI}\%=\frac{a-b}{a}\times 100$ (19).

a = Mean number of trophozoites in the negative control wells
b = Mean number of trophozoites in the test wells at sub-MLC concentration

At the end, the MLCs of the examined agents were confirmed by negative culture of the exposed parasites (17).

### Identification of essential oil compounds

Composition of the essential oil was identified by retention indices and comparing their mass spectra with those of the library according to gas chromatography (GC) and GC-mass spectrometry (GC-MS). GC analysis was performed using a Thermoquest gas chromatograph with a flame ionization detector on DB-5 column (60 m × 0.25 mm; film thickness 0.25 μm). GC-MS analysis was performed similarly by GC column using gas chromatograph coupled to a TRACE mass spectrometer (15).

### Data analysis

Data were presented as MLC, GI% and mean of the values. Data analyses were performed with SPSS statistical software, version 16. The mean of MLC values compared with Friedman’s test and *P* value less than 0.05 was considered as statistically significant difference.

## Results

**Fig. 1:**
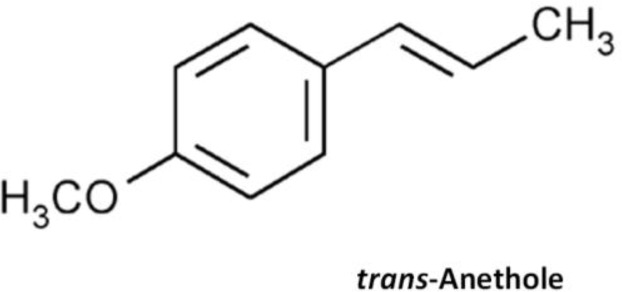
The **s**tructure of the main essential oil component of *F. vulgare* seeds

The in vitro susceptibility assay of the natural products showed that the examined agents had potent activity against *T. vaginalis*. The antitrichomonal activities were time- and concentration-dependent. The hexanic and methanolic extract of *F. vulgare* were the most effective agents against the five *T. vaginalis* isolates with MLC of 400 and 360 μg/ml after 24 and 48 hours incubation, respectively (Fig. 1). The lowest effect was related to the aqueous extract of *F. vulgare* and the essential oil and anethole had the same antitrichomonal properties with MLC of 1600 μg/ml (Table 1, 2). Growth inhibitory of the natural products was observed at sub-MLC concentrations. The GI% was between 75% to 99% after 24 and 48 hours of exposure (Table 1, 2). The *T. vaginalis* isolates were sensitive to metronidazole with a mean MLC of 13.7±6.8 μg/ml (for 24 hours of incubation) and 6.8±3.4 μg/ml (for 48 hours of incubation). Susceptibility variation was detected among the *T. vaginalis* isolates so that the Tv3 isolate was more sensitive to the some natural products than to the others.

**Table 1:** Efficacy of the essential oil and extracts of *F. vulgare* and anethole on five *T. vaginalis* isolates, in comparison with metronidazole, after 24 hours of incubation

***Agents and Drug***	***Lowest MLC (N.^a^)***	***Highest MLC (N.^a^)***	***Mean^b^ MLC***	***Lowest GI%c (N.^a^)***	***Highest GI%c (N.^a^)***	***Meanb ± SD GI%^c^***	**P*-value%^d^***
Essential oil	1600 (5/5)	1600 (5/5)	1600	90 (3/5)	95 (2/5)	92±2.7	
Hexanic extract	400 (5/5)	400 (5/5)	400	90 (2/5)	99 (1/5)	94.4±4.3	
Methanolic extract	400 (5/5)	400 (5/5)	400	80 (1/5)	99 (2/5)	92.6±7.9	
Aqueous extract	12,800 (2/5)	25,600 (3/5)	20,480	99 (5/5)	99 (5/5)	99±0	*P*<0.001
Anethole	1600 (5/5)	1600 (5/5)	1600	96 (1/5)	99 (2/5)	97.8±1.3	
Metronidazole	6.25 (1/5)	25 (1/5)	13.7	90 (2/5)	99 (2/5)	94.6±4.5	

a Number of *T. vaginalis* isolates

b The mean of five susceptibility test values (MLC, μg/ml) conducted on five distinct *T. vaginalis* isolates

c GI% indicates sub- MLC concentration causing a percentage of growth inhibition

d Statistical comparison of the mean of MLCs

**Table 2:** Efficacy of the essential oil and extracts of *F. vulgare* and anethole on five *T. vaginalis* isolates, in comparison with metronidazole, after 48 hours of incubation

***Agents and Drug***	***Lowest MLC (N.^a^)***	***Highest MLC (N.^a^)***	***Mean^b^ MLC***	***Lowest GI%c (N.^a^)***	***Highest GI%c (N.^a^)***	***Meanb ± SD GI%^c^***	**P*-value%^d^***
Essential oil	1600 (5/5)	1600 (5/5)	1600	95 (2/5)	99 (1/5)	96.6±1.8	
Hexanic extract	200 (1/5)	400 (4/5)	360	90 (1/5)	99 (3/5)	96.8±3.9	
Methanolic extract	200 (1/5)	400 (4/5)	360	75 (1/5)	99 (2/5)	92.8±10	
Aqueous extract	12,800(3/5)	25,600(2/5)	17,920	95 (1/5)	99 (4/5)	98.2±1.7	*P*<0.001
Anethole	1600 (5/5)	1600 (5/5)	1600	98 (1/5)	99 (4/5)	98.8±0.4	
Metronidazole	3.1 (1/5)	12.5 (1/5)	6.8	90 (2/5)	99 (1/5)	93.8±3.8	

a Number of *T. vaginalis* isolates

b The mean of five susceptibility test values (MLC, μg/ml) conducted on five distinct *T. vaginalis* isolates

c GI% indicates sub- MLC concentration causing a percentage of growth inhibition

d Statistical comparison of the mean of MLCs

Hydrodistillation of *F. vulgare* gave an essential oil in 2.5% yield (w/w %) relative to dry weight of the plant. The identified constituents representing 98.61% of the total essential oil are presented in Table 3 with retention indices and quantitative result. E-anethole as a phenylpropene was the major compound (88.41 %) in the essential oil.

**Table 3:** Chemical composition of the essential oil from the seeds of *F. vulgare*

***No***	***Compound***	***RI^a^***	***Percentage***
1	α-Thujene	924	0.04
2	α-Pinene^b^	932	0.03
3	Camphene	946	0.02
4	Sabinene	969	0.06
5	β-Pinene	974	0.06
6	Myrcene	988	0.25
7	α-Phellandrene	1002	0.03
8	α-Terpinene	1014	0.05
9	ϱ-Cymene	1020	0.05
10	ο-Cymene	1022	0.04
11	Limonene	1024	0.44
12	1,8-Cineole	1026	1.03
13	Z-β-Ocimene	1032	0.46
14	γ-Terpinene	1054	1.02
15	cis-Sabinene hydrate	1065	0.03
16	Fenchone	1083	2.46
17	Terpinolene	1086	0.05
18	ο-Guaiacol	1087	0.06
19	Linalool	1095	0.26
20	α-Campholenal	1122	0.05
21	Borneol	1165	1.08
22	α-Terpineol	1186	0.06
23	Dihydro carveol	1192	0.06
24	ϱ-Anisaldehyde	1247	1.19
25	Z-Anethole	1249	0.04
26	E-Anethole^b^	1282	88.41
27	Z-Caryophyllene	1408	1.06
28	α-Humulene	1452	0.02
29	γ-Muurolene	1478	0.02
3	β-Bisabolene	1505	0.06
31	Germacrene A	1508	0.01
32	δ-Cadinene	1522	0.04
33	Spathulenol	1577	0.03
34	Caryophyllene oxide	1582	0.07
35	Phenylpropene		88.45
36	Other		10.16
	Total		98.61

a RI, Retention indices relative to C7 – C24 n-alkanes on the DB-5 column

b The identification was also confirmed by co-injection with an authentic samples

## Discussion

In this study, the antitrichomonal activity of *F. vulgare* and anethole was evaluated in comparison with metronidazole, the drug of choice for treatment of trichomoniasis. The susceptibility testing was conducted on five distinct *T. vaginalis* isolates to improve the validity and reliability of the results. Except the aqueous extract of *F. vulgare*, the other natural products exhibited considerable antitrichomonal activity and killed 100% of the trophozoites at their MLC concentrations. After 24 hours of exposure, the most effective agents were the hexanic and methanolic extract at concentration of 400 μg/ml and followed by the oil and anethole at concentration of 1600 μg/ml. After 48 hours, the activity of the hexanic and methanolic extract increased with a mean MLC of 360 μg/ml. Also the mean of metronidazole MLCs for the isolates were 13.7 and 6.8 μg/ml, after 24 and 48 hours of incubation, respectively. The efficacies of the examined agents were statistically significant difference and time- and concentration-dependent. The trichomonacidal property of *F. vulgare* can be due to the presence of potentially antimicrobial compounds such as methyleugenol, estragol (methyl chavicol), anethole and a phenylpropanoid derivative, dillapional (8, 11).

To the best of our knowledge, in vitro anti-protozoal activity of *F. vulgare* has never been studied and there are not data for comparison with our results. But in recent years, significant numbers of studies have documented the antitrichomonal effects of some medicinal plants. The most plant species with considerable antitrichomonal activity are in three families including asteracea, lamiaceae and myrtaceae (20). Avocado, with scientific name *Persea americana*, has been reported as the most effective plant on *T. vaginalis* (21). In this study, the IC_50_ values of the chloroformic and ethanolic extracts of *P. americana* seeds were obtained at concentration of 0.524 and 0.533 μg/ml, respectively, in comparison with IC_50_ at 0.037 μg/ml for metronidazole (21). The effect of avocado was significantly stronger than *F. vulgare*, although the efficacy of avocado was evaluated after 72 hours of incubation. Antitrichomonal property of *Allium hirtifolium* (Persian Shallot) was reported (22). The authors demonstrated high efficacy of hydroalcoholic and dichloromethane extract of *A. hirtifolium* on the parasite at MIC concentration of 10 and 5 μg/ml after 48 hours of exposure, respectively (22). Organosulfur compounds like allicin, ajoene, diallyl sulfides and alliin are related to antimicrobial properties of *Allium* species (23).

Another potent trichomonacidal plant is *Ocimum basilicum*. Essential oil of *O. basilicum* inhibited growth of *T. vaginalis* (100%) at concentrations of 30, 20 and 10 μg/ml after 24, 48, and 96 hours, respectively (24). The main component of *O. basilicum* essential oil is linalool and it probably should be an effective antitrichomonal natural compound. In addition to the spermicidal property of *Sapindus saponaria* at concentration of 2.5 mg/ml, antitrichomonal activity of this herb has been reported by Damke and colleagues (25). In this study, the antitrichomonal activity of water-ethanol and butanolic extract of *S. saponaria* was investigated on two distinct isolates. The extracts inhibited the growth of the clinical isolate at concentration of 0.156 mg/ml but the other isolate (ATCC strain) had MIC of 0.312 mg/ml for the water-ethanol extract and 0.156 mg/ml for the butanolic extract (25). The susceptibility variation between the two isolates is well in accordance with our findings. The in vitro sensitivity variation of *T. vaginalis* isolates to drug and natural products has already been reported (17, 26). The aqueous extract of *Verbena* sp. was only the extract caused death of 100% of the seven *T. vaginalis* isolates at concentration of 4.0 mg/ml but the other plant extracts exhibited various effects on the applied isolates (26).

Other notable antitrichomonal plant that have been reported are alcoholic extract of *Verbascum thapsus* (IC_50_=39.17 μg/ml, after 24hrs), *Eucalyptus camaldulensis* (IC_100_=60 μg/ml, after 72 h), and *Xanthium brasilicum* (IC_100_=500 μg/ml, after 192 h) (27–29).

Investigation of antiprotozoal activity of some phytochemical compounds has exhibited potent activity against *T. vaginalis*. Among the phytochemical compounds, terpenoids, alkaloids, saponins and glycosides had remarkable antitrichomonal activity (20). Hederagenin, a natural derivative of *Cussonia holstii*, has been shown as an effective antitrichomonal agent at concentration of IC_50_ (2.8 mM) after 72 hours of incubation (30). Other phytochemicals with prominent antitrichomonal activity are saponins derived from *S*. *saponaria* with MIC of 0.078 mg/ml after 24 hours of exposure (25).

In the present study, there was no access to clinical drug-resistant isolates, so we do not have any information on the effect of the investigated natural products on the isolates mentioned.

## Conclusion

Regardless of this limitation, the extracts and essential oil of *F. vulgare* and its bioactive component can be considered as a significant antitrichomonal agent. Therefore further in vivo studies are required to be fully assessed their effects and toxicity of the agents. However, theirs low solubility in water is a major challenge of its clinical application.
